# A long term physical and biogeochemical database of a hyper-eutrophicated Mediterranean micro-estuary

**DOI:** 10.1016/j.dib.2019.104809

**Published:** 2019-11-15

**Authors:** Yair Suari, Ayelet Dadon-Pilosof, Tal Sade, Tal Amit, Merav Gilboa, Sarig Gafny, Tom Topaz, Hadar Zedaka, Shira Boneh, Gitai Yahel

**Affiliations:** a Faculty of Marine Science, Ruppin Academic Center, Michmoret, Israel; b Porter School of Environmental Studies, Tel Aviv University, Tel Aviv, Israel; c Dept. of Soil and Water Sciences, Faculty of Agriculture, Food and Environment, The Hebrew University of Jerusalem, Rehovot, Israel

**Keywords:** Micro-estuary, Eutrophication, Marine, Levantine, Coastal

## Abstract

Ruppin's Estuarine and Coastal Observatory (RECO) is a Long-Term Ecological Research station positioned on the East Mediterranean shoreline between Tel-Aviv and Haifa, Israel. We present a comprehensive online database and an accompanying website that provides direct access to the physical, chemical, and biological characteristics of the local coastal marine ecosystem and the Alexander micro estuary. It includes three databases that are updated continuously since 2014: a) In situ stationary sensors data (10 min intervals) of surface and bottom temperature, salinity, oxygen and water level measured at three stations along the estuary. b) Monthly profiles and discrete biogeochemical samples (surface and bottom water) of multiple parameters at four stations located at the inland part of the estuary. Measured parameters include concentrations of chlorophyll*-a*, microalgae and bacteria (counted with a flow cytometer), Nitrate, Nitrite, Ammonium, Phosphate, total N, total P, particulate organic matter (POM), total suspended solids (TSS), biochemical oxygen demand (BOD), as well as Secchi depth in each station c) Bi-weekly profiles, chlorophyll-*a* concentrations and cell counts at two marine stations adjacent to the estuary, (1, and 7 Km from the estuary mouth, at bottom depths of 8 and 48 m). The database also includes historical data for the Taninim micro-estuary (2014–2016). The RECO observatory provides a unique data set documenting the interaction of highly eutrophicated estuarine water with the ultra-oligotrophic seawater of the Eastern Mediterranean. This combination results in sharp gradients of salinity, temperature, dissolved oxygen, and nutrients over very small scales (centimeters to meters) and therefore offers an important data set for the coastal shelf research community. The data set also provide a long-term baseline of the estuary hydrography and geochemistry with the hope to foster effective science-based management and environmental planning of this and similar systems.

**Table:** Specifications Table

Subject	Environmental Science (General)					
Specific subject area	*Long-term database of physical, chemical and biological water properties of a hyper-eutrophic micro-estuary and the adjacent coastal water*					
Type of data	Tables					
How data were acquired	1. *Sampling campaigns for CTD, chemical and biological properties.*					
	2. *Laboratory analysis of surface and bottom water for water chemistry and biology*					
	3. *Continuous sensor sampling for temperature, salinity, oxygen and water level.*					
	*Detailed protocols are provided in the website protocols section*					
Data format	Raw and plots					
Parameters for data collection	Data are intended to provide a good temporal and special coverage of biogeochemical parameters in the estuary.					
Description of data collection	*The data are divided into three separate databases and data collection campaigns.*					
	A. *Stationary sensors Data.*					
	B. *Monthly surveys of water physical and biogeochemical properties along the estuary.*					
	C. Bi-weekly surveys of water physical and biogeochemical properties at marine stations facing the estuary mouth B and C Data are available as an Ocean Data View (ODV) ready table that is updated on a monthly basis (http://reco.ruppin.ac.il/eng/template/?Pid=26).					
Data source location	Alexander estuary, Israel. Sampling stations:					
	**Station**	**Description**	**Sampling type**	**Lon**	**Lat**	**Bottom Depth (m)**
	A5	Alexander outfall	Monthly	34.865	32.396	∼ 0.2
	A4	Michmoret Bridge	Monthly, Sensors	34.869	32.393	∼2.4
	A3	Mid Estuary	Monthly, Sensors	34.891	32.386	∼1.6
	A2	Maabarot	Monthly	34.905	32.368	∼0.3
	A1	Estuary Head	Monthly, Sensors	34.908	32.367	∼0.2
	S1	Deep marine station	∼Biweekly	34.802	32.423	48
	S2	Fish cage station	∼Biweekly	34.834	32.412	32
	S3	Shallow marine station	∼Biweekly	34.858	32.402	7
Data accessibility	*All data are freely available for research and educational purposes. Other usages require written permission from the author*					
	*Data are directly accessible through the project website.*					
	A. *Sensors data sensors data can be plotted or downloaded here (*http://reco.ruppin.ac.il/eng/sensor/)					
	B. *Inland estuarine stations data can be accessed here: (*https://docs.google.com/spreadsheets/d/1-VLXmOMOggn9lV7SBsMzbjeVaFEdWHEINmV1WL0D0t0/edit?usp=sharing*)*					
	C. *Marine stations data can be accessed here: (*https://docs.google.com/spreadsheets/d/1dPFwHSzlA6y6NRV4k1hLH39wZ7hGTjSvUHyinhBMV-s*)*					
	*Since the monitoring is an on-going process, the data is stored and distributed using a dedicated website (*http://reco.ruppin.ac.il/*). Monthly and weekly survey data are provided in google sheet format (to download select “download as” from the file menu). To download or plot continuous sensor data use the self-explanatory interface on the link (*http://reco.ruppin.ac.il/eng/sensor/*)*					
Related research article	Y. Suari, T. Amit, M. Gilboa, T. Sade, M.D. Krom, S. Gafni, T. Topaz, G. Yahel, Sandbar breaches control of the biogeochemistry of a micro-estuary, Front. Mar. Sci. 6 (2019) 224. doi: https://doi.org/10.3389/fmars.2019.00224

**Table:** 

**Value of the Data** • Micro-estuaries with a surface area of less than one square kilometer are very abundant and are expected to become more abundant due to global warming and increased water utilization • This five-year time series represents typical processes in highly eutrophicated micro-estuary in semi-arid environments • This database can be used to study the effect of environmental conditions on the biogeochemistry of a eutrophicated micro-estuary • The marine database can be used to characterize the climatology along the east Mediterranean continental shelf • Managerial changes planned for the estuary might make this dataset a dataset of reoligotrophication [1]

## 1 Data

**Fig. 1:**
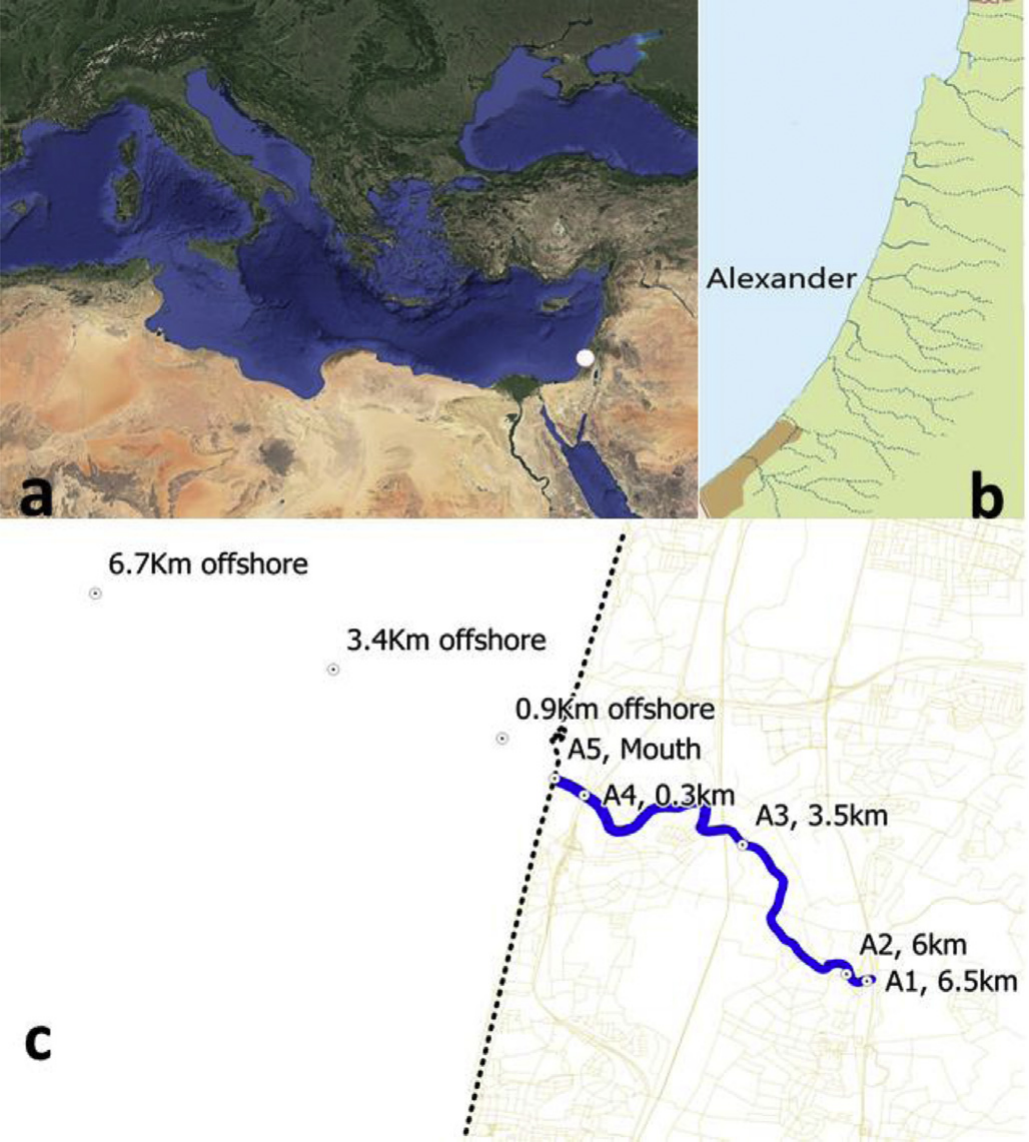
The location of (a) The Alexander estuary at the Mediterranean Levantine basin denoted by white dot. (b) Coastal streams along the Israeli coast and specifically, the Alexander. (c) Sampling points (black dots) along the Alexander estuary (in blue) and near sea. Position of both marine and estuarine sampling stations is given in kilometers from coastline (The exact station positions are given in the data sheets at the website, http://reco.ruppin.ac.il/eng/template/?Pid=26).

The data we publish at the RECO website originate from a long-term, multi-parameter monitoring program that covers physical, chemical, and biological water properties at several stations along a Levantine micro-estuary and its neighboring coastal sea. A summary of the measured parameters, sampling frequencies, and locations is presented in Table 1 and Fig. 1.

**Table 1:** Index and meta-data for data collected during monitoring of the Alexander an available at the RECO website.

Parameter	Type	Sampling type	Sampling location	Sampling intervals
Temperature + Salinity	Physical	Moored sensor	A1, A3, A4 Surface + bottom	10 min
Water Level		Moored sensor	A4	10 min
Temperature + Salinity		Profiles	A1, A2, A3, A4	Month
Temperature + Salinity		Profiles	S1, S3	∼Two weeks
Dissolved Oxygen	Geochemical	Moored sensor	A1, A3, A4 Surface + bottom	10 min
Dissolved Oxygen		Profiles	A1, A2, A3, A4	Month
Dissolved Oxygen		Profiles	S1, S3	∼Two weeks
Optical Backscatter		Profiles	A1, A2, A3, A4	Month
Optical Backscatter		Profiles	S1, S3	∼Two weeks
Secchi Depth			A1, A2, A3, A4	Month
Suspended Solids		Discrete, Surface and bottom	A1, A2, A3, A4	Month
Particulate Organic			A1, A2, A3, A4	Month
Particulate Inorganic			A1, A2, A3, A4	Month
PO_4_			A1, A2, A3, A4	Month
NH_4_			A1, A2, A3, A4	Month
NO_2_			A1, A2, A3, A4	Month
NO_3_			A1, A2, A3, A4	Month
Chl. *a* Fluorescence	Biological	Profiles	A1, A2, A3, A4	Month
Chl. *a* Fluorescence		Profiles	S1, S3	∼Two weeks
Extracted Chl. *A*		Profiles	A1, A2, A3, A4	Month
Extracted Chl. *A*		Profiles	S1, S3	Week
Fecal Streptococcus		Surface	A1	Month
Fecal Coliform		Surface	A1	Month
Total Bacteria CFU		Surface	A1	Month
Phytoplankton		Surface + bottom	A1, A2, A3, A4	Month
Bacteria count		Surface	A1, A2, A3, A4	Month
Bacteria		Surface	S1, S3	∼Two weeks
Synechococcus		Surface	S1, S3	∼Two weeks
Picoeukariotes		Surface	S1, S3	∼Two weeks
Prochlorococcus		Surface	S1, S3	∼Two weeks

### 1.1 The data are divided into three separate databases

A. In situ stationary sensors data located 0.2 m below the surface and above the bottom at three stations along the estuary (near the head, at the middle and near the mouth). These sensors record temperature, salinity, dissolved oxygen, and water level at 10 minutes intervals. Sensors are retrieved, serviced and calibrated at least monthly.
B. Monthly surveys of water properties along the estuary. These surveys include water column CTD profiles and data that was obtained from discrete water samples (Table 1).
C. Bi-weekly surveys of water properties at two marine stations ∼1 and 6.6 Km from the estuary mouth at bottom depths of 8 and 48 m. These surveys include water column CTD profiles and data that was obtained from discrete water samples (Table 1).

## 2 Experimental design, materials, and methods

### 2.1 Stationary sensors

A stationary array of moored sensors was used to measure temperature and salinity using DST CT salinity & temperature loggers (Star Oddi) and dissolved oxygen concentration that were initially measured with U26-001 data loggers (HOBO, Onset) and later with RBR Solo DO loggers (RBR). The sensors were positioned at three stations along the estuary. Where bottom depth was deeper than 1 m, the array consisted of two sets of sensors, one for surface water and one for the “deep” water (∼2 m). The bottom water sensors were connected to a cable and were kept near the bottom by a lead weight. The surface sensors were connected to a float which held them at ∼20 cm below the water surface. The cable carrying the sensors was inserted into a perforated plastic tube canister to protect it from fouling, vandalism and objects carried by the flow. All sensors were programmed for 10-min measurement intervals and were serviced and calibrated monthly or more frequently when needed. Retrieval/deployment cycle took few hours, and then the sensors were returned to the exact same position. Retrieved data were inspected within few days after retrieval and after a quality check were uploaded to the online database.

### 2.2 Marine sampling

An effort was made to conduct the marine surveys on a weekly basis, but sea conditions and sampling logistics resulted in a sparser sampling scheme, roughly biweekly. Sampling was initially (starting January 2014) made at three stations in front of the Michmoret anchorage, 1.1–6.6 km seaward of the estuary mouth at bottom depths of 8, 30 and 48 m but due to the high similarity of the 30 and 48 m stations and the proximity of the 30 m station to a fish farm, we stopped the sampling at the 30 m depth station at May 1st^,^ 2017. Marine surveys were conducted using a small skiff. Vertical profiles of temperature, salinity, oxygen concentration, chlorophyll-*a* fluorescence, and optical backscatter were measured using a SeaBird, SBE19Plus V2 CTD equipped with a dissolved oxygen sensor (SBE43, Seabird) chlorophyll fluorometer (Cyclops 7, Turner Design) and an optical backscatter sensor (OBS3+, Campbell Scientific). Seawater was collected using a 5 L Niskin bottle (Model 110B, OceanTest) at 10 m depth. Water were collected onboard into dark BOD glass bottles and kept in a dark cool box until they were filtered or preserved in the lab within 3 hours from collection. Samples were preserved for extracted Chlorophyll-*a* (300 ml duplicates) and flow cytometry counts (1.8 mL) as described below.

### 2.3 Estuarine sampling

Water Sampling was conducted monthly along the estuary during the last week of the month, starting from January 2014. Both physical and chemical parameters were measured in each station. Vertical profiles of temperature, salinity, oxygen concentration, chlorophyll*-a* fluorescence and optical backscatter were measured using the same CTD configuration described for marine sampling. Discrete water samples were taken in each station using a horizontal water sampler (5L Niskin bottle, Model 110B, OceanTest Equipment). Where and when water depth exceeded 0.5 m, samples were taken from surface water (∼20 cm below surface) and deep water (∼20 cm above bottom) otherwise, only one water sample was collected.

Water samples for inorganic nutrients (Nitrate, Nitrite, Ammonium, and Phosphate) were drawn directly from the sampler spigot into a disposable syringe and filtered at the field through a 0.2 μm syringe filter (32 mm, PALL Acrodisc). Samples for biological oxygen demand (BOD), total and organic suspend matter, and chlorophyll*-a* were collected into dark bottles. All samples were kept in a cool box on ice. Upon arrival to the laboratory, within 3 hours after collection, 10 mL of the water was filtered onto glass fibers filters (25 mm, Whatman GF/F) for chlorophyll*-a* analysis. Similarly, samples for TSS and POM were filtered (normally 100–200 mL) on a 47 mm GF/F filters (Whatman). Chlorophyll samples and nutrient samples were kept frozen in −20 °C until further analysis.

Samples for BOD 5 were aerated to saturation (at least one hour), diluted to 1:5 ratio with air saturated double distilled water (DDW), transferred into 330 mL BOD bottles and analyzed using a YSI 5100 (YSI) according to the standard method (SM-5210).

### 2.4 Laboratory analysis

#### 2.4.1 Marine samples analysis

##### 2.4.1.1 Flow cytometry counts

Flow cytometry was the standard method used to quantify total concentrations of the microbial community in the seawater. Samples (1.8 mL) were fixed with Glutaraldehyde (EM grade, 50%) to final concentration of 0.1% (0.4% for estuarine samples), incubated for 15min at room temperature, frozen in liquid nitrogen, and stored in −80 °C until further analysis with flow cytometry. An Attune® Acoustic Focusing Flow Cytometer (Applied Biosystems) equipped with a syringe based fluidic system and 488 and 405 nm lasers, was used to measure the concentration and cell characteristics of non-photosynthetic microbes and the three dominant autotrophic groups in the Mediterranean waters: *Prochlorococcus*, *Synechococcus*, and eukaryotic algae. Taxonomic discrimination is based on orange fluorescence of phycoerythrin and red fluorescence of Chlorophyll [5], side-scatter (a proxy of cell volume [2], and forward-scatter (a proxy of cell size, [3,4]. Each sample is analyzed twice. First, for determination of ultra-phytoplankton with the discriminator (threshold) set on the red in both the blue and violet lasers. Next, a second run is used to analyze cells with no autofluorescence, using the nucleic acid stain SYBR Green I and a threshold set on the green in both lasers. Reference microspheres were used as an internal standard in each sample and all cellular attributes were normalized to the beads.

##### 2.4.1.2 Chlorophyll-a extraction:

The samples (300 mL duplicates for marine stations and 10 mL for estuarine stations) were prefiltered using 100μm net to remove large zooplankton and benthic debris and filtered onto a Whatman GF/F filter. Filters were kept frozen at −20 °C until processing. To insure efficient Chlorophyll*-a* extraction from hardy coastal and estuarine algae we used the Dimethyl Sulfoxide (DMSO) extraction method of Burnison [5], with small modifications. Briefly, after samples filtration, the glass fiber filters were placed in 20 mL glass scintillation vials with Teflon lined screw caps. Two mL of DMSO (reagent grade) was added and the vials were incubated at 60 °C at dark for 20 minutes. The vials were cooled to room temperature, and 4 mL of buffered aqueous Acetone (90% Acetone, 10% saturated MgCO_3_ solution) were added to the vials. The vials were vortexed and left for all precipitation to sink. Fluorometric readings were taken using a Trilogy fluorometer (Turner designs). Chlorophyll concentration was read using the non-acidification fluorometric method [6] on a Turner Designs Trilogy fluorimeter calibrated using chlorophyll standards (Sigma C6144). The hot DMSO extraction method was tested at the study sites against the standard oceanographic method of cold extraction in 90% acetone and was found to preform similarly on open sea samples but was up to 10 folds more efficient in extracting chlorophyll from estuarine and some nearshore samples (Yahel, unpublished data).

#### 2.4.2 Estuary water analysis

Nutrients determination was carried out manually following standard methods [7]. Starting from January 2016, duplicates of certified reference material (Supelco QC3179) was analyzed with every batch of samples. Briefly, water for nitrite analysis were diluted X10 and nitrate samples were diluted X100 with prior to analysis with double distilled water (DDW). Nitrate and nitrite concentration was determined after reduction to nitrite on a cadmium-copper column. The nitrite produced reacts with sulfanilamide in an acid solution. The resulting diazonium compound is coupled with N-(I-Naphthyl)-ethylenediamine dihydrochloride to form a colored azo dye, the extinction of which was measured spectrophotometrically [8]. The precision of the method was estimated at ±14 (8%) μmol L^−1^ for nitrate and ±2.5 μmol L^−1^ (10%) for nitrite based on the variability of triplicates taken in each sampling session.

Phosphate concentration was also measured spectrophotometrically following the molybdate blue method [9] after x10 dilution with DDW. The precision is estimated as ±1.5 μmol L^−1^ (±4%).

Ammonium concentration was determined using a modified version of the Holmes fluorometric protocol [10] as described in Supplement 1 of Meeder et al. [11] after X1000 dilution with DDW. Briefly, the method uses a stable working reagent, ortho-phthalaldehyde (OPA) that forms a fluorescent complex with ammonium. To account for the variability of the estuarine water that results with highly variable matrix effect, an internal calibration curve was produced for each water sample by spiking known concentration of ammonium standard solution to 3 of the four 2 mL aliquots drawn from each water sample that is being analyzed. After all samples were spiked, 0.5 mL of OPA solution was added to each aliquot and samples were incubated at room temperature in the dark for 4 hr. Fluorescence of the OPA-ammonium complex was read using the ammonium channel of an Aquafluor fluorometer (Turner Designs). Corrections for background fluorescence were determined by measuring the fluorescence on additional aliquots immediately after the addition of OPA. The precision is estimated as ±14 μmol L^−1^ (±4%).

Total and particulate nitrogen, and phosphorus were determined using standard methods [7] (4500-N B. and 4500-P J) respectively. Briefly, 15 mL of sample water (for total) and the GF/F filters (for particulate) were oxidized with persulfate solution at 120 °C for 50 minutes. After digestion, the digestion product was diluted with DDW (1:10 for phosphate and 1:100 for nitrate). Then, the Nitrate and Phosphate concentration in the digested solution was determined using the phosphate and nitrate methods that were described earlier. For the purpose of particulate and total measurements, a calibration curve was measured using increasing dGTP (Guanosine 5′-triphosphate, C_10_H_16_N_5_O_14_P_3_ PCR grade) concentration.

Chlorophyll*-a* concentration was determined as described for the marine samples above with precision estimated as ±14 μmol L^−1^ (±14%).

Total suspended solids and particulate organic mater were measured using the standard methods after minor modifications (ASTM 2540-B and 2540-E respectively) briefly, before sampling, filters (Whatman GF/F) were burned at 300 °C for two hours and weighted, then, sampled water were filtered until the filters were clogged. The filters were dried at 60 °C for 24 hours and weighted again. TSS concentration was calculated using equation (1).

(1) $$TSS\;=\frac{M_{a}-M_{b}}{V}$$

where, *M*_*a*_ is the dry filter mass after sampling, *M*_*b*_ is the filter mass before sampling, and *V* is the volume of sample water that were filtered. Later, the filters were ignited in furnace for four hours at 450 °C. POM concentration was calculated using equation (2).

(2) $$POM=\frac{TSS-M_{i}}{V}$$

where *M*_*I*_ is the filter mass after ignition. Particulate inorganic matter (PIM) was calculated by subtracting POM from TSS.
